# Application of six sigma process to implement the infection control process and its impact on infection rates in a tertiary health care centre

**DOI:** 10.1186/1753-6561-5-S6-P228

**Published:** 2011-06-29

**Authors:** R Rao

**Affiliations:** 1 Microbiology, Apollo Hospital ,Hyderabad ,Ap.India, Hyderabad, India

## Introduction / objectives

Health care sectors are facing major challenges in the form of Hospital Aquired Infections(HAI). Using six sigma we can analyze the problem, come to practical solutions and implement and sustainable improvements.

## Methods

We introduced six sigma in our health care setup for implementation of infection control practices and studied its impact infection rates. After training the identified team in six sigma process we identified the matrices which will be included in the process. The baseline data taken was the lowest rates achieved in the previous year and the goal was to achieve 1/3^rd^ of that rate. All the steps starting from Define to Control phase were done and we could achieve a remarkable decrease in our Infection rates. Various initiatives like Ventilator Associated pneumonia Green Star Project implementation all Intensive Care Units (ICU) Daily checks for implementation and compliance of infection control protocols,empowermentwas extensively carried out. Tools like dashboard,signages,infection control week were used to disseminate information.

## Results

We could achieve Zero SSI rates in six months, Hand Wash compliance to 69% and achieve the target in all device related infections rates. Indirect measurement of number of beds available for new patient, early discharge/shift out of patient and patient satisfaction provided the necessary data to convince all the staff the need to sustain this new initiative.

## Conclusion

This represents the first successful application of Six Sigma corporate performance-improvement method impacting purely clinical outcomes. HAI reduction was highly substantial and sustained after other traditional strategies had failed.

## Disclosure of interest

None declared.

